# Beyond the mouse: organoids, spheroids, and organs-on-chips as the (inevitable) future of malaria research?

**DOI:** 10.1186/s12936-026-05980-3

**Published:** 2026-06-08

**Authors:** Jérôme Dormoi, Rémy Amalvict, Bruno Pradines

**Affiliations:** 1 Unité Parasitologie et Entomologie, Département Risques Vectoriels, Institut de Recherche Biomédicale des Armées, Marseille, France; 2 Aix-Marseille Univ, SSA, AP-HM, RITMES, Marseille, France; 3 IHU Méditerranée Infection, Marseille, France; 4 Centre National de référence du paludisme, Marseille, France

**Keywords:** Malaria, Micro-physiological system, Mouse, *Plasmodium*, Organs-on-chips (OOCs), Organoids, Spheroids

## Abstract

Malaria remains a major global health threat, causing substantial clinical and socioeconomic burdens in endemic regions. Despite important advances in control strategies, progress is increasingly challenged by antimalarial drug resistance, widespread insecticide resistance, diagnostic escape, and adaptive changes in vector behavior. In this context, robust experimental models are essential to improve understanding of parasite biology, host–pathogen interactions, and therapeutic responses. Murine malaria models remain central to preclinical research because they are accessible, genetically tractable, cost-effective, and well suited to controlled studies of immunity, pathophysiology, transmission, and drug efficacy. Continuous refinement of these models through parasite and host genetic engineering, including transgenic parasites and modified or humanized mouse strains, has further expanded their experimental value. However, murine models do not fully reproduce human malaria. Key interspecies differences in parasite biology, antigenic variation, cytoadherence, tissue tropism, placental structure, blood–brain barrier interactions, and disease kinetics limit direct translation, particularly for severe, cerebral, placental, and relapsing malaria caused by *Plasmodium falciparum* and *Plasmodium vivax*. These limitations underscore the need for complementary human-relevant platforms. Micro physiological systems are emerging as a critical addition to the malaria research toolbox. Spheroids offer scalable three-dimensional models for medium-throughput studies; organoids reproduce essential structural and functional features of human tissues such as the liver, brain, placenta, and vasculature; and organ-on-chip systems incorporate flow, shear stress, and multicellular interfaces to better model sequestration, endothelial activation, barrier dysfunction, liver-stage infection, and drug responses. Rather than replacing murine models, these technologies complement them by bridging the gap between reductionist in-vitro assays and human disease biology, while supporting the principles of replacement, reduction, and refinement. This narrative review examines the current landscape of murine malaria models, their major contributions and translational limitations, and the growing importance of micro physiological systems, with their limitations, in next-generation malaria research.

**Graphical Abstract:**

**Figure:**
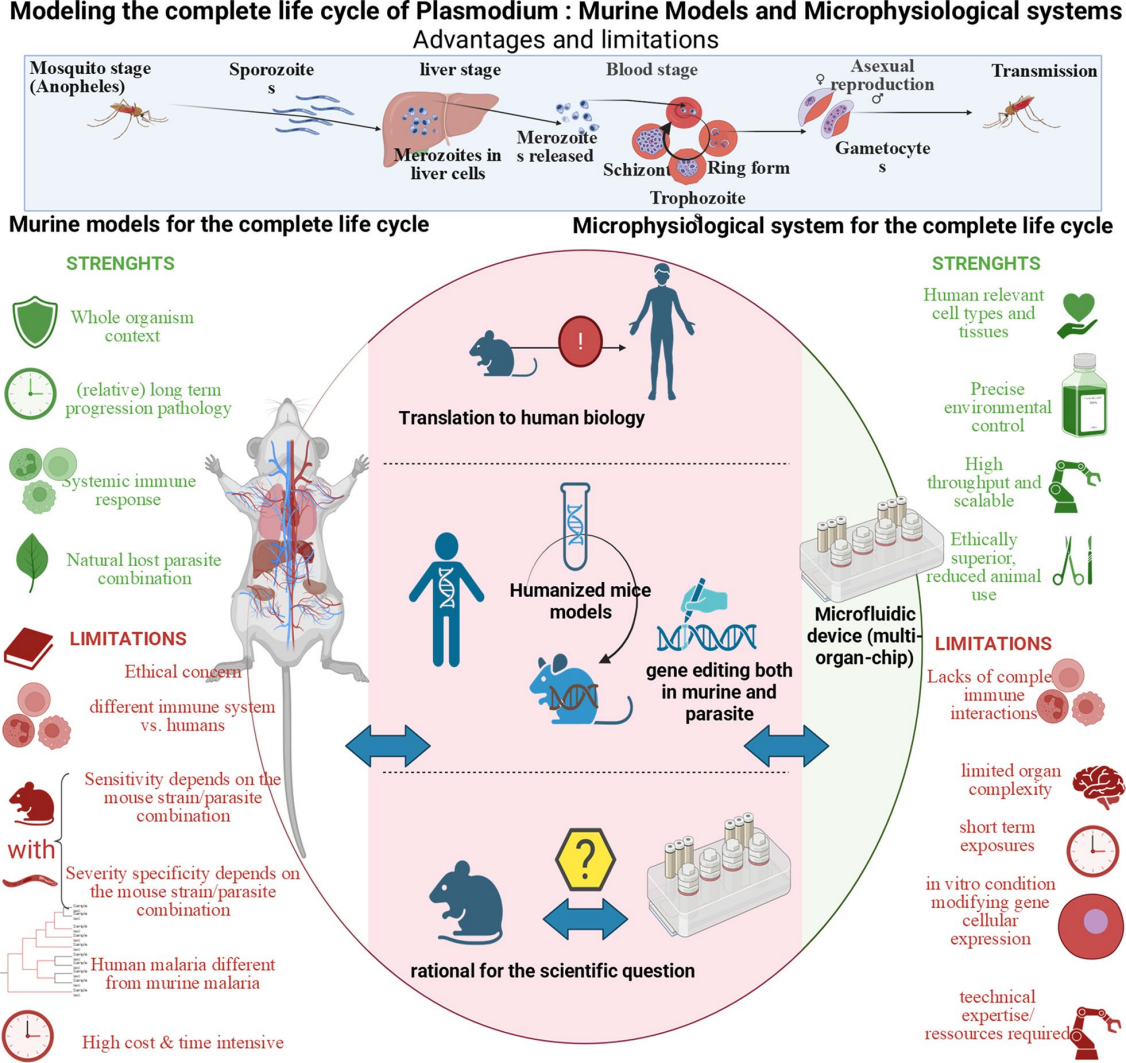

## Background

Malaria is a mosquito-borne infectious disease caused by protozoan parasites of the genus *Plasmodium*, primarily transmitted through the bites of infected *Anopheles* mosquitoes. As of 2024, an estimated 282 million cases were reported globally, resulting in approximately 610,000 deaths [1] The World Health Organization (WHO) African Region disproportionately shoulders this burden, accounting for 95% of all cases and 96% of malaria-related deaths. Children under five remain the most vulnerable, representing nearly 80% of these fatalities [2, 3].

Beyond mortality, malaria is also a significant cause of long-term morbidity, contributing to economic hardship for affected individuals and health systems [4, 5]. Effective elimination of malaria requires addressing eight major challenges: [1] the influence of climate change; [2] growing insecticide resistance; [3] behavioral adaptations of mosquito vectors, especially following the deployment of long-lasting insecticidal nets (LLINs); [4] limitations in the sustainability of current preventive measures; [5] resistance of *Plasmodium* species to antimalarial drugs; [6] the absence of an effective, widely deployable vaccine; [7] the need for improved health education to change risky behaviors; and [8] the socioeconomic factors contributing to disease persistence [6–10].

At least three of these challenges—prevention, drug resistance, and vaccine development—can be effectively investigated using animal models. A wide range of malaria animal models have been established, involving species such as mice, rats, gerbils, chickens, and non-human primates. These models exploit the host specificity of various *Plasmodium* strains. In mice (*Mus musculus*), strains such as *Plasmodium berghei* (*Pb*) and *Plasmodium vinckei* (*Pvk*) are extensively studied. These rodent malaria models provide critical insights into host–pathogen interactions, disease pathophysiology, and therapeutic development. Recent advancements in molecular biology and genetic engineering have expanded the versatility of these models, enabling gene deletions, transgenic parasite lines, and reporter constructs to investigate immune mechanisms and antimalarial resistance [11, 12].

Nevertheless, it is important to recognize that each animal model presents distinct pathological profiles. For instance, *Plasmodium gallinaceum* (*Pg*) in poultry exhibits different clinical features than rodent or primate malaria, even at comparable levels of parasitemia or considering days after infection [13–15]. Similarly, the clinical outcomes of infection may vary depending on both the *Plasmodium* strain and the host mouse strain.

The same *Plasmodium* species may induce different disease phenotypes in different mouse genetic backgrounds, and conversely, the same mouse strain may exhibit distinct responses to various rodent *Plasmodium* species [13]. Stringent animal welfare regulations—such as Directive 2010/63/EU (Europe) [16], the Guide for the Care and Use of Laboratory Animals (USA) [17], the Animals (Scientific Procedures) Act (UK) [18], and corresponding guidelines in South Korea [19] emphasize the ethical treatment of animals in research. These frameworks mandate humane endpoints, enriched environments that support natural behaviors, and a rigorous assessment of scientific necessity.

Despite this regulatory complexity, the mouse remains the most widely used animal model for malaria research, offering a practical and ethically viable platform to explore both uncomplicated and severe disease phenotypes. In this review, we focus on malaria models, with particular attention to their relevance in the study of cerebral malaria, malaria-associated acute respiratory distress syndrome (MA-ARDS), malaria-associated acute kidney injury (MA-AKI), and other systemic complications. We also discuss their role in the evolving field of organoids and organs-on-a-chip (OOC), underscoring the continued importance of animal models in preclinical research landscape.

## Background of mouse models

Rodent malaria models have been instrumental in understanding malaria pathophysiology since their development in the late 1940s [20]. These models gained prominence during World War II, particularly due to the vulnerability of military personnel to *P. vivax* infections in the Pacific theater [21]. Since then, the strategic value of developing effective antimalarial therapies using rodent models has been well established [22].

The discovery of *Plasmodium* sp. in African thicket rats (*Grammomys surdaster*) marked a turning point in experimental parasitology [23]. These species and its derivatives—*Pb*, *P. chabaudi*, (*Pc*), *Pvk and P. yoelii* (*Py*)—remain widely used in malaria research. Each of these species presents unique pathological features in murine hosts, allowing researchers to model both uncomplicated and severe forms of human disease.

A key aspect of translational research lies in the ability of animal models to accurately replicate the pathophysiological characteristics observed in human diseases. In the context of drug development and the evaluation of drug efficacy (antimalarials and vaccines), the immunological similarity between the model species and humans must be carefully assessed [24, 25]. Furthermore, the use of natural parasite-host combinations offers distinct advantages over artificial or non-natural combinations, as they more accurately reflect the co-evolutionary dynamics between the host and the pathogen. These natural systems provide insights into real-world immune responses and disease progression, which may be masked in non-native host models [24–26].

Consequently, selecting an appropriate animal model requires striking a balance between anatomical, physiological, and immunological similarities to the human condition. Ultimately, rigorous validation of the model’s fidelity is essential to ensure that results are transferable to clinical applications in humans [26, 27].

*P. chabaudi chabaudi* AS (*Pcc*AS) is commonly employed in studying uncomplicated malaria due to its self-limiting course, while *P. berghei* ANKA (*Pb*A) is an alternative model for experimental cerebral malaria (ECM) which exposes differences with human cerebral malaria (HCM) pathology. ECM also offers a compromise to largest animal models such as primate malarias (*Plasmodium coatneyi, Plasmodium fragile*) in macaques. *P. yoelii* 17XNL (*Py*17XNL) and *P. yoelii* YM (*Py*YM) strains offer additional insights into reticulocyte invasion and lethal non-cerebral infection, respectively. *P. vinckei petteri* (*Pvp*) and *P. vinckei vinckei* (*Pvv*) provide models for both mild and severe disease phenotypes.

Murine models are further strengthened by advances in genetic engineering. Rodent *Plasmodium* strains have been modified to express genes from other *Plasmodium* species or luminescent markers, enabling real-time tracking of parasite development from mosquito transmission to hepatic and blood stages. These tools also facilitate high-throughput screening of candidate antimalarial compounds [28, 29]. This approach has been demonstrated not only in mouse models of malaria, but also in malaria models in non-human primates (NHPs), such as *Plasmodium cynomolgi* [30], as well as in human malaria parasites [31]. The decision to use all or part of these models depends on the scientific question being studied as well as the methods used to monitor the experiments (molecular biology, MRI, anatomopathological).

Strains resistant to drugs such as pyrimethamine, artesunate, and mefloquine are maintained by the BEI Resources Repository. These resistant lines are invaluable for studying drug mechanisms and testing novel therapeutic strategies.

In sum, murine malaria models offer a reliable and adaptable platform for dissecting disease mechanisms, evaluating drug efficacy, and developing vaccines [32]. Their relevance continues to grow alongside technological innovations in molecular biology and imaging, reinforcing their status as a cornerstone of malaria research.

## Severe malaria in humans

Severe malaria is a life-threatening manifestation of *Plasmodium* infection, particularly caused by *P. falciparum*. It encompasses a range of clinical complications resulting from multi-organ dysfunction, often requiring intensive care. Severe malaria typically evolves from untreated or inadequately managed uncomplicated malaria, and although it represents only 1–3% of clinical cases, it accounts for a disproportionate number of fatalities [33].

Several host and environmental factors influence the likelihood of severe malaria, including endemicity, patient age, ABO blood group, and access to early diagnosis, particularly in urban areas, and treatment [34–37]. Besides *P. falciparum*, severe malaria cases can also be attributed to *P. knowlesi* and *P. vivax*, albeit less frequently [38, 39].

The clinical definition of severe malaria is anchored in evidence of organ dysfunction, which may involve the brain (cerebral malaria), lungs (ARDS), kidneys (AKI), liver, and spleen. Diagnostic criteria include acidosis, hyperparasitemia, hypoglycemia, jaundice, renal impairment, severe anemia, and signs of circulatory collapse [40, 41].

Cerebral malaria (CM) is defined by the WHO as unarousable coma (unable to localize a painful stimulus) in a patient in whom the presence of *P. falciparum* asexual [42, 43]. *Pf*EMP1 (*Plasmodium falciparum* erythrocyte membrane protein 1) is a highly variable surface protein expressed on infected red blood cells. It mediates cytoadherence by binding endothelial receptors such as ICAM-1 (intercellular adhesion molecule) and EPCR (endothelial protein C receptor), allowing infected cells to sequester in microvasculature [44]. In cerebral malaria, this sequestration occurs in brain capillaries, leading to inflammation, impaired blood flow, and blood–brain barrier dysfunction. Specific *Pf*EMP1 variants are associated with severe disease due to their strong binding affinity to brain endothelial receptors. Thus, *Pf*EMP1 is central to the pathogenesis of cerebral malaria [44, 45].

ARDS manifests as difficulty breathing due to pulmonary inflammation and fluid accumulation, with radiographic evidence of pulmonary edema and oxygen desaturation (< 92%). It affects 20–30% of severe malaria patients and is associated with high mortality despite intensive care interventions [46–48].

AKI in malaria is defined by elevated creatinine and urea levels, reduced urine output, and systemic fluid imbalance. The pathogenesis involves hemodynamic instability, hemoglobinuria, direct parasitic damage, and secondary infections. AKI is often underdiagnosed but represents a major prognostic factor [49].

Severe malaria also affects hematological and metabolic systems, leading to severe anemia (hemoglobin < 5–7 g/dL), coagulopathy, hepatic dysfunction (elevated bilirubin), and hypoglycemia (blood glucose < 40 mg/dL). In extreme cases, it progresses to multi-organ failure, including splenic rupture and retinopathy, with features such as papilledema and retinal whitening [50].

Understanding the complexity of severe malaria pathophysiology requires integrative models that replicate the disease’s clinical heterogeneity and molecular underpinnings—an endeavor in which murine models have proven indispensable.

## Experimental cerebral malaria (ECM)

Among the severe manifestations of malaria, cerebral malaria (CM) is one of the most fatal and complex syndromes. To study its underlying mechanisms, the *Pb*A model has emerged as the gold standard for inducing ECM in susceptible mouse strains, especially C57BL/6 and CBA/J [5].

ECM recapitulates many clinical and neuropathological hallmarks of HCM, including neurological impairment, blood–brain barrier disruption, and leukocyte infiltration. The development of ECM is influenced by both host and parasite factors. In mice, ECM typically manifests between days 4 and 6 post-infection, with death occurring before day 11 in most cases [51–55]. Not all mouse strains are equally susceptible to ECM (see Table 1). C57BL/6 and SJL/J are highly susceptible, whereas BALB/c, CBA and DBA/2 are more resistant [51, 56–58]. The breding phenotyphe does not seem to play a role as Swiss Webster (outbred) and FVB (inbred) are both sensitive to *Pb*A- ECM [53, 57]. In the *Pb*A model, the onset and penetrance of neurological manifestations are sensitive to experimental parameters, including host strain/age/sex, parasite line, and the initial parasite burden [59–61]. For example, ECM incidence is significantly higher in C57BL/6 mice injected with *Pb*A-infected erythrocytes from genetically similar donors, highlighting the importance of host-parasite compatibility [56]. In general, higher inocula accelerate parasitemia rise and may compress the pre-symptomatic phase, whereas lower inocula tend to delay the appearance of neurological signs and can increase inter-animal variability in the timing of disease. Moreover, malaria rodent parasite exposes a 22 to 24 h cycle. Most studies do not specify the stages of the parasite infecting mice [62, 63] excepted if the aim is the study of synchronous malaria parasite in blood in the framework of host circadian rhythm. While *Pc* maintains his synchronous population *Pb remains* asynchronous independently of circadian rhythm host [64, 65]. Our experiments with the murine model of cerebral malaria did not allow us to draw conclusions about the importance of the parasite's stage, but rather about the injected dose and the number of parasites per unit volume. The different methods for synchronous malaria parasites remain for in vitro or ex vivo studies [66–69].

**Table 1:** Combination between laboratory rodent strains and *Plasmodium* species

		*P. berghei*	*P. chabaudi*	*P. vinckei*	*P. yoelii*
		ANKA/KSP 11 K173 NK65/ NK65-E	*adami* (DK, DS) *chabaudi* (AS, AJ, BC, CB, DK, ER)	*brucechwatti* *lentum* *petteri* (AR, AS, BS, CR, HW) *vinckei* (ATCC30091, CY)	17XL 17XNL N67 N67C YM
Mice					
129/Sv	IB	ECM pos [55]	Cellular/humoral immunity [200] (knockout mice)	Susceptibility phenotyping (F1 descendance from mixed background BL6 × 129sv mice) [201]	Immunology and vaccination study [202]
A/J	IB	Cellular/humoral immunity [203] drug Assay [204] Cellular/humoral immunity [53]	Pathology comparison [205]	Cellular/humoral immunity [206] vaccination [207]	Comparative studies of cellular/humoral immunity, vaccination, drug assay [208, 209] comparative studies of cellular/humoral immunity [210]
BALB/c	IB	Cellular/humoral immunity in pregnant mice [211] non-ECM model [5, 51], and Vaccine test [212] ECM intermediate [55] Splenomegaly [142]	Cellular/humoral immunity [206]	Parasitemia between different rodents [23]	Antimalarials antibody [213] vaccination [214]
C3H	IB	ALI/ ARDS [91] ECM intermediate [55] Vaccination [117]	Vaccination [215]	Cellular/humoral immunity [206]	Cellular/humoral immunity, severe malaria model [151] Genome comparison in rodent malaria pathogenesis [216]
C57BL6 and C57BL10	IB	AKI (NK65) [105] blood-stage phenotype characterization [217] ECM pos [5, 51, 55] Placental malaria [218] Splenomegaly [142]**,** ARDS [90]	Cellular/humoral immunity [206] ARDS treatment [89] AKI [106] Non-ECM model [5] Spleen damage and treatment [146] Vaccination [219]	ND	Genome comparison in rodent malaria pathogenesis [216]
CBA	IB	Cellular/humoral immunity [220], ECM neg (= *Pb*K173) [58] ECM pos (= *Pb*ANKA) [5, 53]	Cellular/humoral immunity [220]	Cellular/humoral immunity [221] comparative genomics of phenotypes [222]	Immunology [223] Comparative studies of cellular/humoral immunity [208] Antimalarials [224]
CD1	OB	Splenomegaly [142] Vaccination [225]	Acquired mutation of resistance to antimalarials [226]	Comparative genomics of phenotypes [222] drug assay [227]	Pharmacological screening, safety/toxicity testing [228] Vaccination [229]
DBA/2	IB	ALI/ ARDS [230] ECM neg [56]	Survival comparison between mice and *Plasmodium* strains [231]	ND	Comparative study of infection and immune response [232]
FVB	IB	ECM pos [57]	ND	ND	ND
NMRI	OB	Metabolism [233]	vaccination [219]	Drug assay [234]	Drug assay [235]
OF1	OB	Cellular/humoral immunity [203]	drug assay [236]	Drug assay [237]	ND
SKH1	OB	Blood-stage phenotype characterization [217]	ND	ND	ND
Swiss Webster	OB	Blood-stage phenotype characterization [217] Drug assay [238] ECM pos [53], cardiac pathology [122] genomics [239]	Cardiac pathology [122] Trans gestational maternal malaria infection [240]	Cellular/humoral immunity [241] Drug assay [242] Blood-stage phenotype characterization [243]	Pharmacological screening, comparative pathological studies drug assay [244]
Rat					
Brown Norway	IB	Cellular/humoral immunity [245]	ND	Only reports concerning adaptation from wildlife to laboratory condition [246]	ND
Lewis	IB	Cellular/humoral immunity [247] Organ damages/ ECM pos [82] Anemia [248]	ND	Anemia [248]	ND
JCL/ Sprague–Dawley	OB	ECM pos (young) [80] Drug assay [249]	Cellular/humoral immunity [250]	ND	Infection model [251]
Wistar	OB	Drug assay (young) [252, 253] Metabolism [254]	ND	Host adaptation/virulence regulation [255]	ND
WM/M	IB	ECM pos [81]	ND	ND	ND

Murine strains mostly experienced in laboratory are listed in vertical while the different *Plasmodium* strains are listed in horizontal. The intersection exposes the main experimental uses in this combination. ECM: model for cerebral malaria, pos: fully expressing feature of ECM, intermediate: the rate and the expression of cerebral damage are partial. Cellular/humoral immunity: study of immune response, blood-stage phenotype characterization: comprehensive study of parasitic cycle about duration invasion and cell host. Drug assay: efficacy test of antimalarial candidates, vaccination: vaccine procedure and humoral response (young) mean animal age before weaning (21 days old). ND: no data

Likewise, inoculation route [70, 71] can influence early kinetics: intravenous administration delivers parasitized erythrocytes directly to the circulation and is often associated with more synchronous infections, while intraperitoneal delivery can introduce a delay due to absorption and may broaden the onset window. Consequently, the “ECM window” for cerebral readouts (e.g., intravital imaging, BBB permeability assays, temperature drop and neurological assessment) is best defined by physiological and parasitological staging (parasitemia dynamics and early clinical signs) rather than by days post-infection alone, particularly when comparing studies that use different doses or routes [52, 70–73].

Neurological assessment of ECM includes clinical scoring systems such as SHIRPA (SmithKline Beecham, Harwell, Imperial College, Royal London Hospital, Phenotype Assessment) and SNAP (Simple Neuroassessment of Asymmetric Impairment) [52, 71–75]. These tools facilitate the objective quantification of neurological symptoms, such as limb paralysis, ataxia, and altered behavior.

Although ECM models share several features with HCM, key differences remain [76, 77]. In humans, cerebral malaria (HCM) is classically linked to *P. falciparum*–infected erythrocyte sequestration within the brain microvasculature, mediated by *Pf*EMP1 variants binding endothelial receptors such as ICAM-1 and EPCR. This adhesive phenotype promotes microvascular obstruction, endothelial activation (e.g., upregulation of adhesion molecules), dysregulated coagulation/protein C signaling, and blood–brain barrier dysfunction, culminating in vasogenic edema and, in fatal pediatric cases, marked brain swelling [78, 79]. In contrast, ECM in susceptible mouse strains infected with *Pb*A reproduces key downstream features (endothelial activation, barrier leakage, edema and microhemorrhages), but pathogenesis is more strongly shaped by immunopathology, notably the recruitment and intravascular retention of activated leukocytes (especially CD8+ T cells) that damage the neurovascular unit and alter cerebral hemodynamics following an inflammation process [72]. Recent studies have expanded the ECM model to include young and older rats (e.g., Sprague Dawley), which offer larger brain size and enhanced imaging capabilities. These adaptations further underscore the model’s versatility and relevance for translational research [80–82].

Accordingly, although murine ECM has been invaluable for dissecting inflammatory cascades and testing mechanistic hypotheses, important limitations constrain its use as a direct surrogate for clinical cerebral malaria studies. These include major species and parasite differences in cytoadherence biology (lack of the extensive *P. falciparum* var/PfEMP1 repertoire and human-specific receptor usage), differences in the relative contribution of parasite sequestration versus T cell–driven pathology, distinct cerebral vascular/venous anatomy and blood–brain barrier properties, and the fact that many interventions protective in mice have not translated into effective adjunctive therapies in humans [26]. Furthermore, preliminary studies have highlighted differences between the mechanical aspect of CD36-mediated HCM, while experimental cerebral malaria has been observed in CD36-/- mice [83]. Thus, ECM is best viewed as a complementary model capturing selected components of HCM pathophysiology rather than a fully faithful platform for predicting human cerebral clinical outcomes [26]. More broadly, we reiterate the conclusions of the work by Langhorne et al*.* Murine models allow us to improve our knowledge and thy are also useful in eliminating ineffective therapeutic trials (vaccines or curative treatments) [84].

## Malaria-associated acute lung injury (ALI) and malaria-associated acute respiratory distress syndrome (ARDS)

Murine models of malaria-associated acute lung injury (MA-ALI) and acute respiratory distress syndrome (MA-ARDS) have emerged as valuable tools to investigate pulmonary complications that mirror aspects of severe malaria in humans [85]. Although no animal model fully recapitulates the entire spectrum of human ALI/ARDS, rodent systems offer crucial mechanistic insights under controlled experimental conditions [86].

Several *P. berghei* strains—such as *Pb*K173, *Pb*NK65-E, and *Pb*A—can induce MA-ALI and MA-ARDS in mice, depending on the host strain used [87–89]. In contrast, *Pb*NK65-NY does not typically result in significant pulmonary pathology. MA-ARDS is characterized by pulmonary edema, pleural effusion, alveolar hemorrhage, and impaired gas exchange, reflected in reduced blood oxygen saturation. Histopathological hallmarks include infiltration of neutrophils and monocytes, increased vascular permeability, and alveolar epithelial damage [90].

Host genetic background plays a critical role in disease severity. C57BL/6 and DBA/2 mice are generally more susceptible to MA-ARDS, while BALB/c mice display relative resistance (see Table 1). Notably, DBA/2 mice infected with *Pb*K173 provide a robust model of lung injury without cerebral involvement, making them particularly useful for studying MA-ALI/ARDS in isolation. It was the first model for MA-ALI [91].

*Pb*NK65-E exhibits a pronounced initial tropism for reticulocytes, the immature red blood cells, during the early stages of murine infection. This preference is a defining characteristic that shapes its initial growth dynamics and pathogenesis. As the infection progresses and the host's pool of circulating reticulocytes becomes depleted (either through parasite consumption or a suppressed erythropoietic response) the NK65-E strain undergoes a critical phenotypic shift, significantly increasing its invasion of mature normocytes to sustain its parasitemia [88]. In stark contrast, the *Pb*A strain demonstrates a fundamentally different invasion strategy from the outset, displaying a much higher intrinsic capacity to invade reticulocytes when they are abundant [92]. The study by Vandermosten et al*.* explicitly demonstrates that the development of malaria-associated acute respiratory distress syndrome (MA-ARDS) is critically dependent on this parasite-host combination and coincides precisely with the phase of predominant normocyte invasion [88]. Therefore, the pathogenicity of NK65-E in models of MA-ARDS is intrinsically tied to the timing of its shift from a reticulocyte-preferring to a normocyte-invading phenotype. Failing to account for this dynamic, stage-dependent tropism can lead to significant misinterpretations of virulence mechanisms. Accurately reflecting on this temporal shift is essential for understanding the divergent pathological pathways of these two widely used *Pb* strains [85, 92, 93]. In experimental models of *Pb* infection in mice, the onset of clinical symptoms associated with MA-ARDS typically manifests between 7 and 12 days post-infection, with mortality commonly occurring around day 20 [85, 87, 91]. This temporal progression is not uniform across all parasite-host systems; rather, it is highly contingent upon specific biological variables, most notably the parasite strain employed, and the size of the inoculum administered. For instance, the *Pb*NK65-E strain—unlike the ANKA strain, which primarily induces cerebral complications—elicits severe pulmonary pathology characterized by leukocyte infiltration, microhemorrhages, and vasogenic edema, culminating in fatal respiratory failure [85, 87, 88, 94]. The precise timing of symptom emergence and lethality is directly influenced by the parasite’s erythrocytic tropism: the shit from reticulocytes to normocyte invasion led to the development of MA-ARDS. Higher inoculum doses (1 × 10⁶ iRBCs or higher) [95] can accelerate parasitemia kinetics, thereby advancing both symptom onset and time to death (with consistent pulmonary involvement), while lower doses (1 × 10^4^ iRBCs) [96] may delay or attenuate disease severity. Consequently, studies reporting disease timelines without specifying strain characteristics or inoculation parameters risk generating non-reproducible or misleading conclusions about pathogenesis. To ensure scientific rigor and cross-study comparability, future work must explicitly detail these critical experimental variables, as underscored by data linked to previous data [85, 87], which align with the findings that MA-ARDS progression is exquisitely sensitive to the parasite-host combination and invasion [94, 95].

Beyond histology, molecular investigations have revealed an accumulation of neutrophils producing chemokines and myeloperoxidase, contributing to oxidative stress and tissue injury. A decrease in the activity of the epithelial sodium channel (ENaC) has been noted, which impairs fluid clearance and promotes edema [85, 96]. These findings are consistent with other inflammatory and degenerative lung pathologies. Moreover, a recent study has shown both an increase of IL-10 and an alteration of lung microbiota as triggers for ALI/ARDS in *Pb*A infected DBA and C57BL6 mice [97]. Although not fully analogous to the human condition, murine models of MA-ALI and MA-ARDS remain essential for dissecting immunopathological pathways, identifying potential biomarkers, and evaluating anti-inflammatory therapies in severe malaria. Interestingly, discrepancies exist between human and murine responses. For example, CD36-mediated sequestration plays a major role in murine lungs and brain but is not a dominant mechanism in humans. Moreover, while MA-ARDS in mice correlates with high parasitemia, human ARDS often occurs independently of parasitemia levels, particularly in pediatric cases. The expression of adhesion molecules (e.g., ICAM-1, VCAM-1) and inflammatory mediators like heme oxygenase-1 (HO-1) is elevated during MA-ARDS. HO-1 has a protective effect by reducing endothelial activation and leukocyte adhesion, thereby mitigating lung injury [98]. Even if the clinical phenotype remains close to that of humans, cellular and molecular characteristics of animal species relevant to modeling ALI/ARDS can be discussed. Indeed, rodents share only pulmonary intravascular macrophages with Humans, while their percentage identity with human hypervariable region of TLR4 remains low (48%), with high nitric oxide production and difference in chemokines and chemokines receptors [99–101].

## Malaria-associated acute kidney injury (MA-AKI)

MA-AKI is a frequent and severe complication of *Plasmodium* infection, especially in *P. falciparum*-induced malaria. In murine models, MA-AKI can be effectively studied using *Pb*A and other rodent *Plasmodium* species such as *Pcc*AS and *Py* [102–105]—see Table 1. These models have provided valuable insights into the renal manifestations of malaria and the mechanisms underlying parasite-induced nephropathy.

Mouse models for MA-AKI typically employ C57BL/6 [106], BALB/c, ICR and CD-1 strains, infected with doses ranging from 1 × 10^3^ to 2 × 10^7^ *Pb*A-infected erythrocytes in 200 μL of sterile saline solution [102–104, 107, 108]. BALB/c mice are particularly useful for renal studies, as they are less susceptible to cerebral complications, allowing for a focused investigation of kidney pathology.

MA-AKI is characterized by elevated levels of creatinine and urea, reduced glomerular filtration rate, and structural damage to renal tubules and glomeruli [109]. These signs are often accompanied by hematuria, proteinuria, and changes in urine color and odor. Urine output may initially decrease (oliguria or anuria) before transitioning to a phase of dark, concentrated urine, reflecting acute tubular necrosis and impaired filtration capacity [102, 110].

The onset and severity of MA-AKI correlate with parasitemia and the host's genetic background. Inflammatory responses play a critical role, including infiltration of mononuclear cells and oxidative stress, particularly in the glomeruli and proximal tubules. Histopathological studies frequently show immune complex deposition, endothelial swelling, and glomerular capillary congestion.

Mice with induced AKI have also been studied in conjunction with traditional ischemia models (e.g., renal pedicle clamping) or chemical nephrotoxins like cisplatin, providing a comparative basis to evaluate malaria-specific renal pathology. Notably, female mice are less susceptible to cisplatin-induced AKI, while male mice exhibit greater sensitivity to ischemia–reperfusion injury [111, 112]. These sex differences are important considerations in model design [113].

An innovative model by Terkawi et al*.* employed clodronate to deplete phagocytic cells in mice infected with *Py*17XNL, converting a non-lethal infection into a severe AKI phenotype [114]. This approach underscores the role of immune modulation in renal outcomes and presents a versatile platform for evaluating immunotherapeutic strategies.

Biochemical assays for MA-AKI in mice require careful sample collection. Metabolic cages are now widely used to separate urine and feces, minimizing contamination and behavioral stress. Biomarkers such as albumin, urea, creatinine, and novel indicators like neutrophil gelatinase-associated lipocalin (NGAL) are employed to assess renal injury [115, 116].

While MA-AKI is often self-limited in some models (e.g., *P. yoelii*), it can be fatal in others (e.g., *Pb*A-infected CD-1 or ICR mice [102, 110]). Its resolution is typically dependent on the reduction of parasitemia and initiation of antimalarial treatment, highlighting its reversibility if managed early. Meanwhile, despite their experimental advantages, murine models of MA-AKI also present important limitations. In *Pb*A infections, vascular accumulation of infected erythrocytes appears to involve, at least partially, CD36-dependent interactions and parasite proteins including schizont membrane-associated cytoadherence (SMAC) protein [117]. However, these mechanisms differ structurally and functionally from the *Pf*EMP1-mediated cytoadherence that characterizes *P. falciparum* infections in humans [118]. In addition, *Pb*A-infected erythrocytes lack the prominent electron-dense “knobs” that facilitate stable endothelial adhesion and microvascular sequestration in *falciparum* malaria. Consequently, several authors preferentially use the terms “vascular accumulation” or “tissue accumulation” rather than true sequestration in *Pb*A, particularly in cerebral and renal microvessels. By contrast, knob-mediated cytoadherence is well established in both *P. falciparum* and the non-human primate parasite *P. coatneyi*, supporting the relevance of these models for studying endothelial dysfunction and microvascular injury [119]. Therefore, while murine systems remain highly valuable for investigating inflammatory and immunopathological mechanisms involved in MA-AKI, caution is required when extrapolating cytoadherence-dependent vascular processes to human malaria.

## Other organs involvements in severe malaria: cardiac, hepatic, and splenic pathology

Beyond cerebral, pulmonary, and renal complications, severe malaria also exerts deleterious effects on the heart, liver, and spleen. Murine models allow detailed analysis of these organ systems, offering insights into pathophysiological mechanisms and systemic consequences of *Plasmodium* infection.

### Cardiac pathology

Myocardial involvement in malaria is increasingly recognized in both clinical and experimental settings. In murine models, especially in *P. falciparum*-infected humanized BALB/c mice [120], cardiac dysfunction is often associated with inflammatory cell infiltration (lymphocytes and monocytes) [120] while *P. falciparum* disrupts myocardial architecture in humans [121]. Both in rodent *Plasmodium* infected mice and humanized *P. falciparum*-infected mice, Stanley Imade et al*.* and Toledo-Marelli et al. confirm inflammatory cardiomyopathy [120] and a reduction in pro-resolving lipid mediators [122], crucial for tissue repair [122]. These disturbances impair cardiac contractility and may contribute to circulatory failure.

In both humans and mice, parasitic antigens like glycosylphosphatidylinositol (GPI) may act as cardiotoxins, inducing cardiomyocyte apoptosis [123]. Nevertheless, heart failure is rarely the primary cause of death in murine malaria models; it typically occurs secondary to dysfunction in brain, lungs, or kidneys [91, 124]. Murine models, combination of fatal *Pb* or self-resolving *Pc* in C57BL/6 [124], BALB/c [125], ICR [126], and C3H/z [91] mice have all been used to explore cardiac effects, although no universally accepted model for malaria-induced cardiomyopathy currently exists.

### Liver pathology

The liver is a central organ in malaria pathogenesis, serving both as an initial site of sporozoite development and a target of systemic inflammation during the blood stage [127, 128]. Malaria-associated liver pathology (MALP) in mice is commonly modeled using *P. berghei* (ANKA or NK65) or *Pc*AS in susceptible strains such as C57BL/6 [129, 130].

Both in human and mice, hepatic manifestations include hepatomegaly, hepatocyte ballooning, mononuclear infiltration, hemozoin deposition, and sinusoidal congestion. Histological examinations reveal puffy hepatocytes, Kupffer cell hyperplasia, and focal necrosis. Biochemical indicators such as elevated alanine transaminase (ALT) and aspartate transaminase (AST) are used as markers of hepatocellular injury [131]. Although hepatic involvement is increasingly recognized as an important component of malaria pathogenesis, current experimental evidence relies predominantly on murine blood-stage infection models rather than true pre-erythrocytic liver-stage systems. In particular, *Pb*A infection in C57BL/6 mice, has also demonstrated significant hepatic injury associated with parasite burden, inflammatory infiltrates, oxidative stress, and fibrosis independently of CD8+ T-cell-mediated pathology [132, 133]. Additional studies using BALB/c, C57BL/6, or ICR mice infected with *Py* or *Pc* further highlighted the contribution of neutrophils, low-density granulocytes, and type I interferon pathways to liver inflammation and tissue remodeling during malaria infection [134, 135]. More recently, spatial and single-cell transcriptomic analyses provided detailed characterization of host–pathogen interactions within the *Plasmodium*-infected murine liver, including hepatocyte and Kupffer cell responses [136].

However, these murine models present important translational limitations. Rodent parasites such as *Pb*, *Py*, and *Pc* differ substantially from human parasites (*P. falciparum* and *P. vivax*) with respect to erythrocyte tropism, sequestration behavior, parasite biomass, and immune activation. Moreover, murine hepatic pathology develops rapidly and is strongly influenced by mouse genetic background, which complicates extrapolation to human disease. Human studies have demonstrated that even uncomplicated malaria may induce significant liver dysfunction characterized by apoptosis, cholestasis, and NF-κB activation, particularly during *P. falciparum* infection [137, 138]. Importantly, non-human primate models (for example *Saimiri boliviensis*) infected with *P. vivax* reproduce several histopathological features observed in humans and may therefore complement murine systems for translational studies [139].

Advanced models use genetically modified or immunodeficient mice (e.g., NOD/SCID) to replicate human immune responses more closely [140, 141]. These systems provide valuable platforms for studying immune-mediated liver damage and the impact of malaria therapies on hepatic function.

### Splenic pathology

The spleen plays a dual role in malaria: it filters parasitized erythrocytes and orchestrates immune responses. In murine malaria models, splenomegaly is one of the earliest and most prominent signs, observable from day 3 post-infection. Enlargement is often accompanied by hematological changes such as lymphopenia, thrombocytopenia, and leukopenia [142]. Inoculum typically ranges from 1 × 10^5^ to 1 × 10⁷ iRBCs [142, 143].

Murine models using *Pb*ANKA [144], *Pb*K173 [142], *Pc* [145–147], or *Pv* and *Py* [148] strains have shown variable degrees of splenic involvement. Histopathological studies reveal red pulp congestion, disrupted white pulp architecture [146, 147], and extensive infiltration by myeloid cells [146, 147]. These alterations contribute to anemia through increased clearance of both infected and healthy erythrocytes. In contrast to human cases, where splenic rupture is a rare but documented complication, this event is not commonly observed in mice [149, 150]. Nonetheless, strain-dependent variability in susceptibility to splenic damage has been reported. The phenotype of resistant/sensitive malaria associated spleen damage murine models is open to discussion. Comparative studies between mouse strains infected with *Pb*K173 demonstrate that ICR mice indeed show more severe splenic atrophy and higher mortality rates, but this does not render C57BL/6 mice "resistant" in any absolute sense. Instead, C57BL/6 mice exhibit a distinct pathological trajectory characterized by preserved gross organ integrity despite significant microscopic and cellular disruption. This pattern reflects their genetic predisposition toward Th1-type immune responses, which effectively control parasite replication but simultaneously drive inflammatory processes that remodel splenic architecture [142]. The misconception of resistance likely arises from methodological limitations in earlier studies that focused primarily on survival outcomes or gross organ weights without detailed histological assessment. Modern techniques employing flow cytometry, immunohistochemistry, and advanced imaging [146, 148, 151] have revealed that even in genetically resistant strains like C57BL/6, malaria infection induces profound changes in splenic microanatomy that affect both immune function and hematological parameters. Therefore, accurate scientific communication should emphasize the qualitative differences in pathological patterns between strains rather than implying categorical resistance, particularly in the context of malaria models where splenic involvement is universal and mechanistically central to disease progression and resolution [142].

Collectively, these organ-specific pathologies emphasize the systemic nature of severe malaria and the need for integrative models. Murine systems continue to be essential for investigating the cascade of immunological, vascular, and metabolic disturbances underlying multi-organ failure in malaria.

Table 1 exposes the different combinations between murine strains and *Plasmodium* strains in murine malaria models.

## Emerging tools: from humanized mouse models to organ-on-a-chip (OOC) technologies

In the pursuit of more accurate and translational models of malaria, the scientific community has increasingly turned to advanced systems such as humanized mouse models, organoids, and OOC platforms. These innovations aim to overcome the limitations of conventional murine models and to recapitulate human-specific responses to *Plasmodium* infection with greater fidelity.

Humanized Mouse Models Humanized mice—immunodeficient rodents engrafted with human hematopoietic stem cells (HSCs), hepatocytes, or tissues—represent a promising approach for studying human *Plasmodium* species, particularly *P. falciparum* and *P. vivax*. These models allow full liver-stage development and erythrocytic cycles of the parasite, enabling in vivo exploration of host–pathogen interactions, immune responses, and drug efficacy [152].

For example, the HIS (human immune system) mouse model facilitates investigation of immune-mediated pathology and vaccine responses [153] (see Table 2).

**Table 2:** Experience in humanized mice models.

		NOD/SCID gamma (NSG), FRG-KO huHep—and other humanized models
*Plasmodium*	*falciparum*	Drug assay [256] Cytometry applied to parasitemia [257]
	*vivax*	*Vivax* malaria cycle in genetically modified C57BL/6 mice [152] Liver stage infection and hypnozoite [154] Liver stage inhibition of *P. vivax* malaria [258]
	*malariae*	ND
	*ovale*	Evaluation of models for quiescent parasites [158]

Humanized models are genetically modified mice which are engrafted with human tissue. In some cases, immune tissue is engrafted on mice giving them a base for human immunological studies. ND: no data

**Fig. 1:**
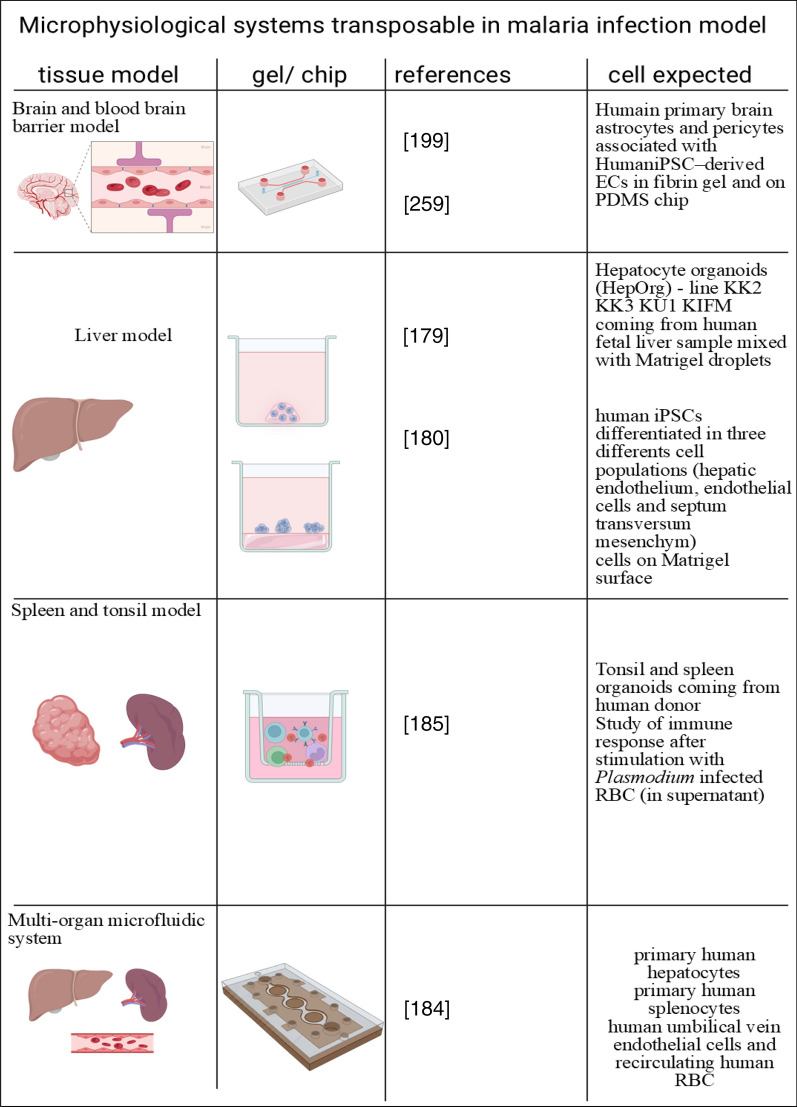
MPS transposed to mimic malaria physiopathology in one or several tissues at the same time. Organs such as brain [259], liver [179, 180, 184], spleen [184, 185] tonsil [185] and endothelial vessel [184] are illustrated in color (BioRender). The MPS is illustrated in the next column, MPS can use adherent cells or organoids mixed or not with Matrigel and supported with a chip. The last column exposes the tissue expected

The FRG KO huHep model (fumarylacetoacetate hydrolase [FAH], Rag2, and IL2rγ knockout mice engrafted with human hepatocytes) supports sporozoite infection and liver-stage development of both *P. falciparum* and *P. vivax* [154]. Humanized hepatocyte-chimeric mice provide a valuable compromise between biological relevance and practical constraints of *in-vivo* animal models, enabling the study of *P. vivax* and *P. ovale curtisi* infections [155], including hypnozoite biology, dormancy, and antimalarial drug efficacy [154]. Notably, the FRG KO huHep model can support the complete parasite lifecycle, encompassing sporozoite invasion, hypnozoite formation, and subsequent reticulocyte invasion [154]. Chimeric mouse models expand the scope of malaria research by reducing animal size and institutional infrastructure requirements whilst maintaining ethical advantages over larger animal systems [156]. However, significant limitations persist: no rodent malaria model currently produces functional hypnozoites of *P. vivax* and *P. ovale curtisi*, and hypnozoite research remains restricted primarily to *Plasmodium cynomolgi* infections in non-human primates, particularly rhesus macaques (*Macaca mulatta*) [157]. This limitation underscores the continued importance of primate models for antimalarial drug development targeting relapsing malaria parasites but also the place of chimeric mice and MPS to address the lack of tools needed to study the hepatic phase of malaria (see Fig. 1).

Despite their potential, humanized models have several limitations: high cost, complex engraftment procedures, and limited scalability. Furthermore, the immunological environment often remains incomplete or non-fully functional, which can affect the generalizability of immune findings.

As you can see, few literature data were found for *P. malariae* and *P. ovale* in humanized mice models (Table 2). Indeed, the literature on these two models remains limited [158] compared to that concerning *P. falciparum* or *P. vivax* [159, 160]. In fact, *P.* ovale and *P.* malariae are difficult to maintain in continuous *in-vitro* culture. For these two species, ex vivo cultures are performed directly from human infections [161, 162]. Furthermore, these two species do not show decreased sensitivity to antimalarial drugs; they are therefore generally well managed and treated [163, 164]. Thus, these factors could explain the lack of data on *P. malariae* and *P. ovale* in humanized mouse models. Moreover, we consider the findings made for hypnozoites in *P. vivax* to be a useful model for understanding hypnozoites in *P. ovale*.

Furthermore *P. knowlesi* is not reported on Table 2. It is clear that in the current context of increasing *P. knowlesi* infections [165], the development of study models is important. *P. knowlesi* can be cultured *in-vitro*, continuously, on human red blood cells, following the work of Armistead et al. [166] and Moon et al. [167]. It can be synchronized using guanidium hydrochloride, although this affects the resumption of the cycle. Gamete transmission from in vitro culture to female Anopheles mosquitoes is also documented [166]. In theory, *P. knowlesi* infection is entirely modellable both in humanized mice models but also in MPS, as 2D or 3D liver tissues and blood cells already exist [168].

Organoids and organs-on-chips (OOCs) are three-dimensional human stem cell–derived systems that mimic the structural and functional characteristics of tissues such as the liver, brain, lung, and kidney. These mini-organs have proven valuable for investigating malaria pathogenesis, especially in models of liver-stage infection [169].

**Fig. 2:**
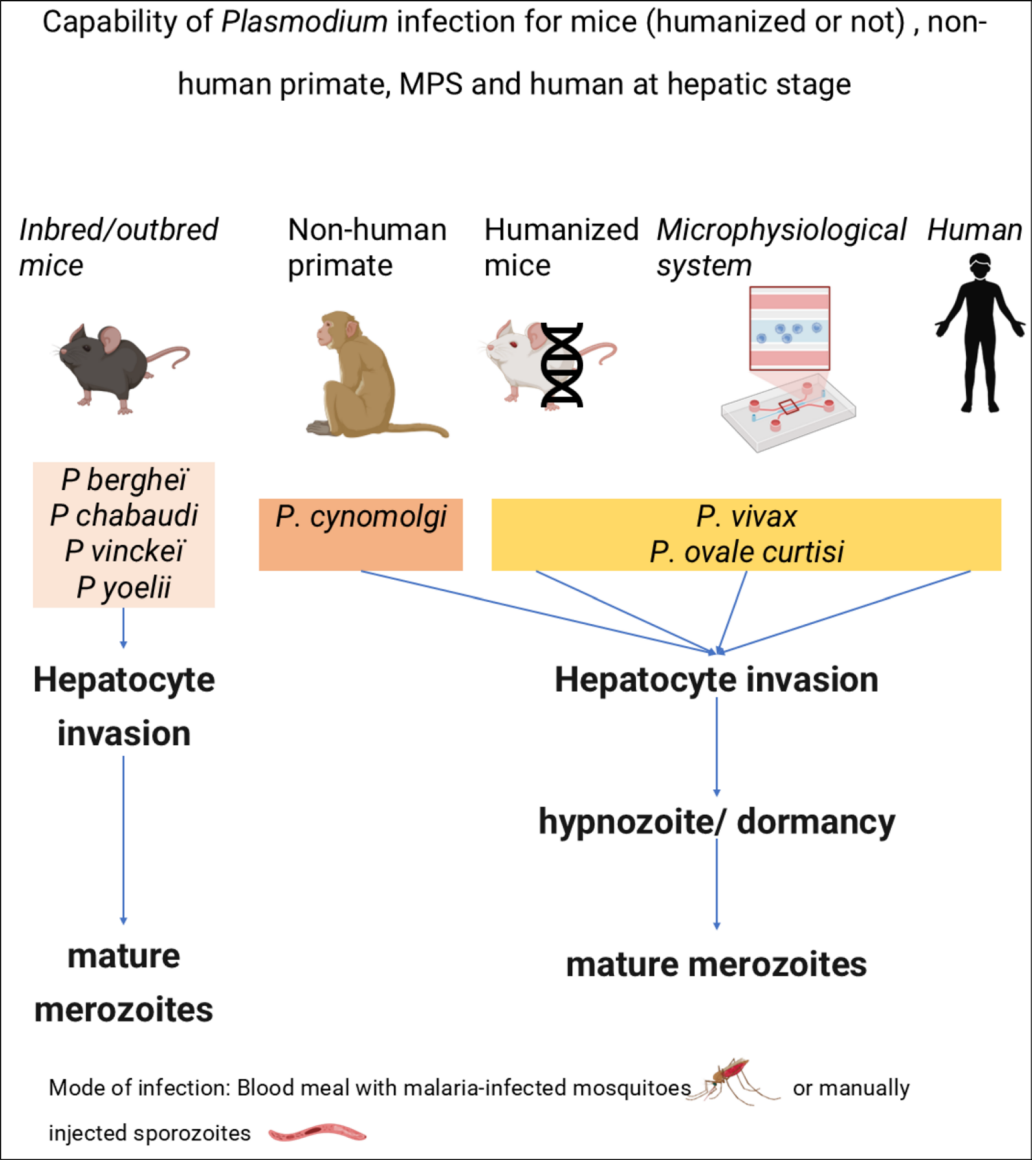
Schematic representation of the model useful to evaluate hepatic stage. Human pathology with hypnozoite and dormancy can be mimic through different systems such as NHP model [157], but also humanized mice [154] and MPS [180], meanwhile there is no hypnozoite in natural murine malaria combination

**Fig. 3:**
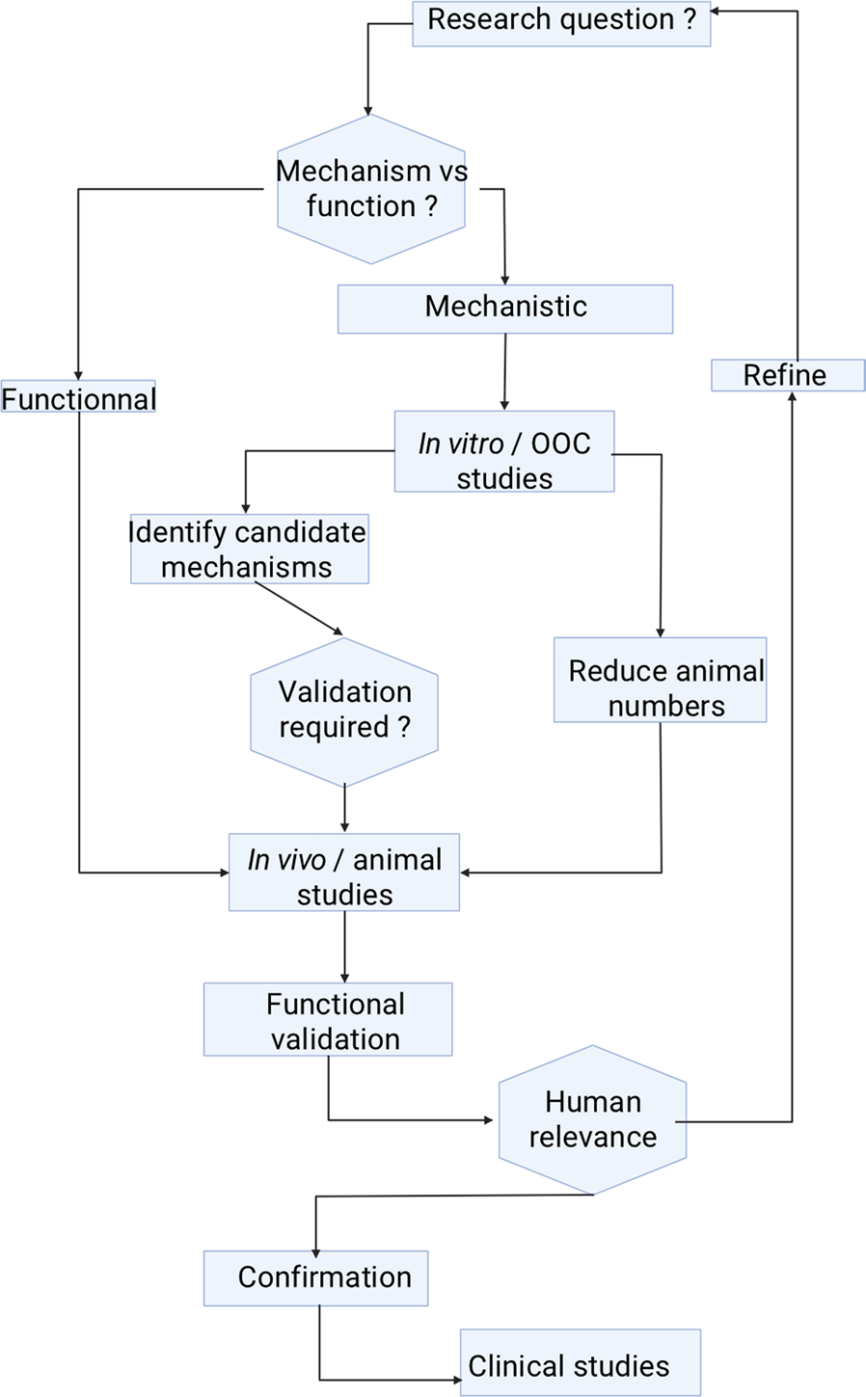
The most robust strategy combines several model systems: OOC platforms for early mechanistic studies, animal models for functional validation, and human studies—including controlled human malaria infection—for final confirmation, with iterative refinement as knowledge advances

Liver organoids, for example, provide an in vitro environment for testing drug efficacy and hepatotoxicity during the hepatic phase of *Plasmodium* infection. Brain organoids, though in early stages of malaria development, may help elucidate the mechanisms of cerebral malaria and neuroinflammation in a human-relevant context [170, 171] (see also Figs. 2 and 3).

OOC integrates microfluidic technology with living human cells to recreate organ-level physiology and mechanical microenvironments. These platforms offer dynamic control, oxygenation, and cell–cell interactions, making them highly attractive for malaria research. Recent developments include liver-on-a-chip models for studying sporozoite invasion and parasite development, as well as blood–brain barrier chip for exploring endothelial dysfunction in cerebral malaria.

## Organs modelling in uncomplicated and severe malaria

The great interest of OOC is to support the culture of primary human cells together in a microfluidic chip. In the framework of malaria infection, several organs are involved, from the brain to the kidney. As previously described, *Plasmodium* infection is a multisystemic illness. Severe malaria is considered when one of the five important organs (brain, heart, lungs, liver, and kidney) is failing. In this paragraph, the actual OOC will be described to know the different capacity of micro physiological systems to support organs involved in *Plasmodium* infections, but also in drug and toxicity screening. We focus on OOC because it is the most advanced system used to support vitro cell culture. These cell cultures can be engineered as a cell monolayer or multilayer [172, 173] but also as three-dimensional cultures like in spheroid [174] or organoid [175].

These platforms should therefore be regarded primarily as complementary mechanistic tools rather than integrated systems capable of reproducing the full complexity of severe malaria pathophysiology. Human in vitro organ models provide valuable insights into endothelial responses, barrier dysfunction, and parasite–host interactions, but they lack the multicellular immune environment that critically shapes inflammation and organ injury during severe malaria. However, their use aligns with the 3Rs principle—Replacement, Reduction, and Refinement—by reducing reliance on *in-vivo* experimentation while increasing human relevance [176].

As organoids and OOC become more sophisticated and scalable, they hold the potential to model inter-organ communication and systemic disease processes, which are currently difficult to replicate in isolated, in vitro or vivo settings. Integration of these systems with murine models can yield a comprehensive, multiscale approach to malaria research, bridging the gap between bench and bedside (Table 1). Recent studies have shown that OOC can be much more than a separate and complementary tool in animal model research, they can be an extension of a protocol initially designed with animals. The ability to transfer murine tissue in the latest system of organ-on-chip highlights not also its usefulness in 3R’s rules but also a more ethical approach in science or in the research process [177]. It is interesting to know OOC can be grafted tissue from human to mouse like Wilson et al. demonstrated in their experience [178]. Even if it is not applicable for malaria models because there is no direct infection between animal and humans, (malaria needs a female *Anopheles* mosquito as vector of transmission) the idea to conciliate both animal and human tissues on the same system remain seducing in the study of zoonosis or viral infection common between human and animal.

Organoids, spheroids, and organ-on-a-chip devices have recently emerged as promising micro physiological systems (MPS) for modelling human–*Plasmodium* interactions, with primary experimental studies demonstrating their capacity to recapitulate key stages of malaria infection. Human liver organoids represent the most advanced application in this field, owing to the liver’s essential role in the pre-erythrocytic cycle [179]. Differentiated fetal hepatocyte organoids support *P. falciparum* sporozoite invasion, maturation, and schizont development, enabling single-cell characterization of host–parasite interactions and the identification of host pathways required for intracellular development. Similarly, iPSC-derived liver organoids permit *P. vivax* infection, capturing both developing exoerythrocytic forms and early merozoite release, marking a significant step toward modelling hypnozoite biology and intrahepatic relapses [180]. Additional work using ductal-cell–derived hepatic organoids has validated these platforms for antimalarial drug testing, demonstrating pharmacological responsiveness of intrahepatic parasites within 3D human tissues [169]. These liver models constitute a clear strength: they provide physiologically relevant, human-specific microenvironments that are not achievable with 2D hepatocyte monolayers and allow mechanistic interrogation of early infection.

In contrast, micro physiological modelling of the symptomatic erythrocytic phase has relied more heavily on organ-on-a-chip systems rather than organoids or spheroids. Microvascular and endothelial chips have proven effective in recapitulating cyto-adhesion, barrier disruption, and mechanical pathology driven by circulating *P. falciparum*-infected erythrocytes. A 3D perfusable human blood–brain barrier model has shown that parasite egress and cytoadhesive interactions disrupt endothelial junctions and activate Janus kinase/ signal transducer and activator of transcription (JAK–STAT) inflammatory pathways, experimentally reproducing aspects of cerebral malaria pathophysiology at high resolution [181]. Mechanical clearance processes relevant to splenic filtration have been reproduced using splenon-inspired microfluidic platforms that physically retain or deform infected erythrocytes, providing experimental access to biomechanical constraints that shape parasite survival [182, 183]. Multi-organ malaria-on-a-chip systems integrating liver, endothelium, and splenic compartments have demonstrated the technical feasibility of maintaining circulating *P. falciparum* infections in interconnected human tissues while assessing both drug efficacy and off-target toxicity [184].

**Fig. 4:**
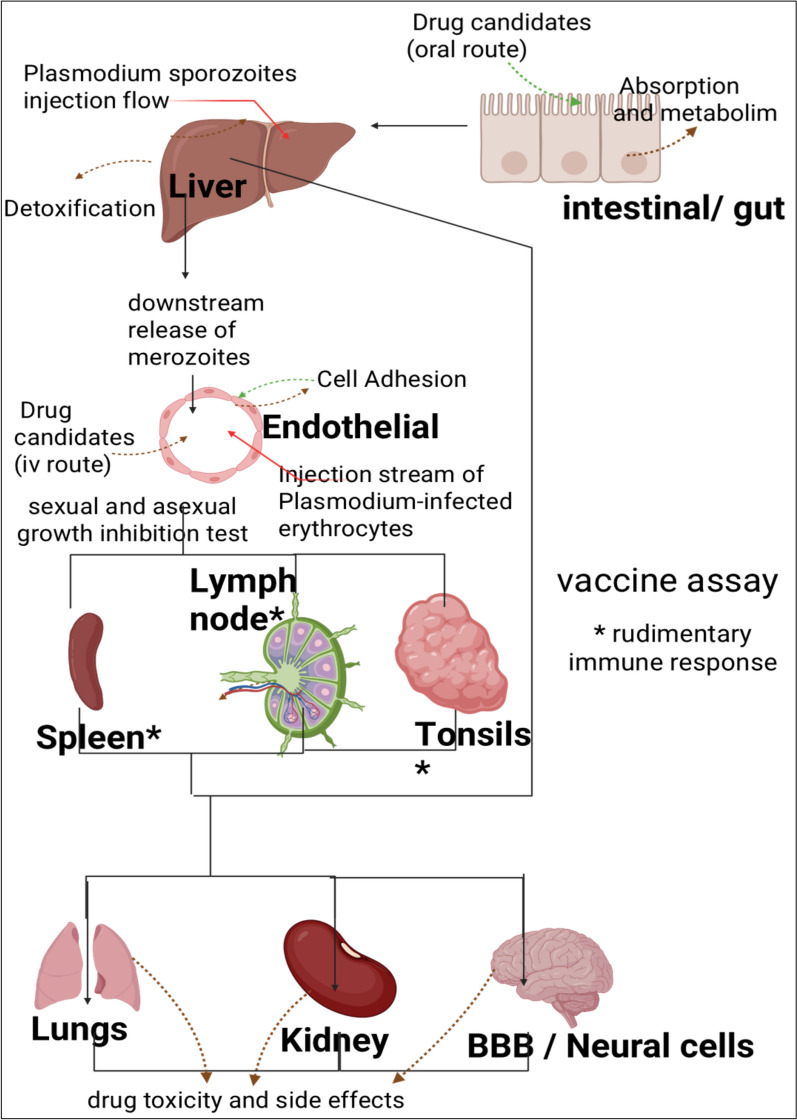
Theoretical and optimized organ on a chip for malaria study. In malaria, several tissues can be involved meanwhile brain, liver and endothelial cell remain the most advanced OOC used to analyze malaria physiopathology. Even if severe malaria remains poor (1% of cases) our knowledge can be enhanced by the utilization of a complex and multi organ-on-a chip to mimic the interaction between *Plasmodium falciparum* (or other species) with human tissue. In our hypothetical OOC, a maximum of nine organ tissues is used with the aim of carrying out not only drug screening but also cellular and rudimentary immune response analysis

Despite these advances, important limitations remain. Liver organoids generally lack vasculature, immune cells, and full metabolic zonation, restricting their ability to capture inflammatory responses or hepatocyte–Kupffer cell crosstalk. Current microvascular and BBB chips model endothelial biology with high fidelity but typically omit perivascular immune components, erythropoiesis, or the complex gradients of cytokines and metabolites present in severe malaria. No published primary study has yet achieved physiologically authentic heart, lung, or kidney infection models, leaving vital organ-specific manifestations of malaria—including acute lung injury, acidosis-associated renal impairment, and cardiac conduction abnormalities—poorly reproduced in MPS platforms (see Fig. 2). Spheroids, while easy to generate and scalable, lack structural polarity and perfusion, and they have not been widely applied to malaria beyond serving as simplified hepatocyte aggregates. Remaining experimental priorities include (1) establishing vascularized, immune-competent liver organoids to study hypnozoite persistence and dormancy; (2) integrating spleen-mimetic filtration modules with endothelial chip to model retention, cytoadhesion, and mechanical clearance simultaneously; (3) developing lung-on-a-chip models directly exposed to infected erythrocytes to probe organ-specific pathology; and (4) expanding multi-organ malaria-on-a-chip systems to capture systemic interactions such as cytokine storm, metabolic disruption, and multi-organ failure. Collectively, primary studies demonstrate that human micro physiological systems can reproduce discrete malaria phenotypes with unprecedented fidelity, yet a fully integrated, system-level MPS model of malaria pathogenesis remains an important and achievable next step for the field. The most advanced MPS systems are based on HUMIMIC Chip4 and 2–5 Organ + from the biotechnology companies named TissUse and Hesperosinc, respectively. These systems allow us to use at least five organ tissues, connected on the same chip. Following this context a hypothetical and optimized model of MPS is proposed on Fig. 4. In our theorical model we use more than 5 tissues (the actual limit even with connected OOC). Red blood cells can be used in various ways within the OoC system. With a peristaltic pump and healthy red blood cells from blood-type donors, we simulate blood flow. This easy-to-use system allows us to model parasite development, from the hepatic stage to merozoite invasion of red blood cells. Another approach involves using infected red blood cells, previously cultured in vitro and then injected into the system. The first approach is more dynamic, as it considers the distance between hepatocytes and red blood cells. In these systems, the infected red blood cells serve both to maintain the infection and to provide antigens for studying the immune response [185]. Obviously, infected red blood cells can also be collected from infected donors.

Evaluation of a vaccine or antimalarial candidate targeting the exo-erythrocytic phase is possible in our optimized theoretical organ-on-a-chip model for malaria studies. However, several constraints must be considered in order to model the immune response or at least provide a preliminary outline.

The use of liver tissue (carcinoma or primary culture) allows for the invasion of sporozoites, but the infectivity rate remains low (< 1.5%) [179]. Given the actual efficacy of whole sporozoite malaria vaccine, there is a scientific rational to study the efficacy of multi-component sub-unit malaria vaccine [186].

Next, one important point is the ability of hepatocytes to present antigen via the MHC complex. To address this, we must look at related disciplines, where the role of the MHC is significant, to determine if existing models are working and what their limitations are. This is the case with non-alcoholic fatty liver disease (NAFLD). In this pathology, hepatocytes affected by NAFLD express an increased number of MHC II receptors [187, 188]. It would therefore be possible to use this type of tissue (primary human hepatocyte) to support a *Plasmodium* liver infection for immunological and vaccine-related purposes. Furthermore, the use of liver carcinoma is also possible. Ma et al. used the HC-04 cell line as a host for *Pb*A or *P.falciparum* infections to study antigen presentation by MHC class I and CD8+ T cell activation [189].

The evaluation of antimalarial treatments at the hepatic stage is simplified by focusing solely on the antimalarial properties of the drugs. In this context, the experience can be analyzed as at the erythrocyte stage, by administering the drug before (prophylaxis) or after (treatment) infection. Yang et al*.* evaluated the efficacy of BLTI (a promising Block Lipid Transport inhibitor) in preventing sporozoite invasion and schizont development [179]. They show that BLT1 significantly decrease schizonts formation.

The in vitro reproduction of the complete *Plasmodium* life cycle remains constrained by the phenotypic and functional characteristics of hepatocyte models. Successful sporozoite invasion requires hepatocytes expressing specific surface receptors such as CD81, which mediate parasite entry. However, the short lifespan and limited proliferative capacity of primary human or murine hepatocytes restrict their experimental utility. To overcome these limitations, immortalized hepatocyte lines have been developed to provide continuous growth and genetic stability over multiple passages, resulting in homogeneous populations suitable for long-term studies. Nevertheless, immortalization often leads to markedly reduced expression of drug-metabolizing enzymes, altered cellular architecture, and the progressive loss of liver-specific functions and intercellular interactions during extended culture [190]. Consequently, despite the availability of immortalized hepatocyte lines that have been used to study the *Plasmodium* hepatic stage, infection rates remain consistently lower than those observed in primary hepatocytes [191]. Among these, the HC-04 cell line has demonstrated a unique capacity to support the *Plasmodium* hepatic stage, offering a promising yet imperfect surrogate model for primary hepatocyte-based systems.

Meanwhile, given the importance of the immune response in malaria infection, the ability of MPS to mimic human immune system is crucial question. Both cellular and humoral response is involved in immune response for malaria infection. These models capture only one part of complex systemic interactions [185]. “Immune response-on a chip” is one of the tissues that exhibits a lack of study and development, leading to scientific discrepancies between the phenotypes observed in patients (or animals) and the responses of MPS. As example, metabolic disruption is defined as the processes leading to adverse effects to major metabolic organs, such as liver or kidney after exposure to environmental, chemical, or biologicals threats and finally it leads to multi organ failure. According to our literature research, metabolic disruption can be reproduced, and it has already been studied in different tissues, such as intestine [192]. Immune responses for organ failure have already been studied by combination of immune cells to tissue-on-chip [193, 194]. Meanwhile, complex immune mechanisms involving cell migration and cytokine storms are not enough developed in organoids or lymphoid node-on-chip [195–197] (Table 3).

**Table 3:** Cells and tissues utilize mimic human malaria infection

Organ modeled	Cell or tissue origin	Cell or tissue obtained/expected	Immortalized lineage	Parameters assessed in relation to the infection	Method assessed in relation to the infection	References
Blood brain barrier	Thermo Fisher Scientific (cat #A18945)	Human episomal iPSCs/reprogrammed CD34+ human umbilical cord blood cells		Heme associated tissue damage	Tissue/cell viability	[170]
					Immunohistochemistry staining	
	University collaboration [260]		HBMEC	Inflammatory response of HBMEC	Immunofluorescence of Brain Organoids markers	[171]
					membrane-based antibody arrays membranes	
					RNA sequencing	
	Cell Systems		HBMEC	Loss of permeability	Immunofluorescent staining of 2D/3D structure	[181]
Brain	ScienCell	HA		Loss of permeability	Microvascular permeability assays	[181]
	University collaboration [261]	Human iPSCs-Neurone, neural progenitor cells		Maturation and differentiation of cells before *Plasmodium* infection	Immunofluorescence of Brain Organoids markers	[171]
					RNA sequencing	
	ScienCell	HBVP		Loss of permeability	Immunofluorescent staining of 2D/3D structure	[181]
					RNA sequencing	
Endothelial vessel	Lonza (Lot #0000636514)	HUVECs		Viability assay	Alamar Test (Thermo Scientific, DAL1025)	[184]
				protein membrane expression	Immunocytochemistry	
Intestine	Small intestine and colon biopsies	Enterocytes, Goblet cells, EEC (*)		Protein membrane expression	Immunocytochemistry	[262] no malaria
Kidney	Kidney biopsies (rat)	Kidney stem cell-derived organoids		Puberulic Acid induced AKI no malaria	Histological and immunofluorescence analyses	[262]
					Real-time RT-PCR	
Liver	Nova Biosis (Lot #BEI)	Primary human hepatocytes		Viability assay	Formazan test	[184]
Lung	Human fetal tissues, Embryonic Stem Cell Line 2, hiPSC lines from fibroblasts	Mesoderm		Swelling, detachment and shedding of infected cells -no malaria	RNAseq and Immunofluorescence staining	bacterial and viral infection/no malaria [263]
		Pulmonary endoderm				
		Branching airway and early alveolar structures				
Lymph nodes	(Animal) lymph node slice	LT, LB		Cytokine diffusion	Optical and fluorescent imaging	[264, 265] no malaria
Spleen	Clinical (organ donor)	Lymphoid follicles (dendritic cells, LT, LB, MΦ)		Inflammatory response markers Vδ2 + T cells Expansion	Surface antibody staining before and after stimulation with iRBC or vaccine candidate	[185, 264]
					RNA sequencing	
	BioIVT (Lot # BRH1392583)	Primary human splenocyte		Viability assay	Alamar Test (Thermo Scientific, DAL1025)	[184]
Tonsils	Clinical (tonsillectomies)	Lymphoid follicles (dendritic cells, LT, LB, MΦ)		Inflammatory response markers and Vδ2 + T cells Expansion	surface antibody staining before and after stimulation with iRBC or vaccine candidate	[185, 264]
					Comparison between patient and organoids for Vδ2 + T cells Expansion	

Lung and lymph nodes remain not fully investigated in malaria research. The different tissues are exposed in this schematic chart as a previous for our theorical and optimized organ on chip. LT: T lymphocyte, LB: B lymphocyte, MΦ: macrophage. Following the different tissues, the origin of cells to generate 2D or 3D structures are exposed with the different experiences carried out to evaluate damages on tissue models

We proposed a rudimentary immune response, based primarily on the production of lymphoid cells as it has already been published, even if the specificity of immune response remains discussed [196, 198]. This theoretical system could be used with appropriate microfluidic pressure to enhance mobility of immune cells. The humoral response will be diffused in all system by the same way [196]. Complementary to our immune response-on-chip, we also consider mechanical phenomena such as clamping and/or patching, which have already been studied in MPS [199].

## Conclusion and perspectives

The continued global burden of malaria, exacerbated by emerging drug resistance, insecticide resistance, and diagnostic challenges, necessitates a robust and multifaceted research approach. While animal models, particularly murine and non-human primate systems, have historically been indispensable for elucidating malaria pathogenesis, immunity, and facilitating therapeutic development, their inherent limitations necessitate a critical re-evaluation and strategic integration with advanced human-based platforms. Murine models, renowned for their affordability, genetic manipulability, and ethical accessibility, have been pivotal in unraveling complex *Plasmodium* biology and evaluating novel interventions. These models have significantly advanced, enabling the study of both uncomplicated and severe malaria manifestations, including cerebral malaria, acute respiratory distress syndrome, severe anemia, placental malaria, and acute kidney injury, thereby providing mechanistic insights crucial for informing clinical strategies. The ability to precisely control experimental parameters and interrogate organ-specific pathology and host-parasite interactions in a manner often difficult or ethically unfeasible in humans underscores their enduring value.

However, the direct extrapolation of findings from murine models to human malaria is constrained by fundamental differences in immune architecture, metabolic pathways, parasite-host interactions, and distinct evolutionary trajectories of rodent malaria parasites compared to *P.falciparum* or *P.vivax*. These disparities, particularly concerning antigenic variation, cytoadherence, tissue tropism, life-cycle kinetics, and human-specific endothelial, placental, splenic, or blood–brain barrier biology, highlight the necessity for a more nuanced application of murine models. The future trajectory of malaria research does not advocate for the abandonment of these foundational models but rather their employment with greater precision: carefully selecting the appropriate model for specific biological questions, explicitly defining the aspect of human malaria being approximated, and rigorously avoiding over-interpretation beyond the intrinsic scope of each system.

This demand for enhanced precision has catalyzed the development and integration of complementary platforms exhibiting greater human relevance. Humanized mouse models offer improved in vivo study of human malaria parasites, while (MPS, including spheroids, organoids, and organ-on-chip devices, are creating novel avenues for reconstructing human tissue environments with increasing fidelity. These advanced platforms hold particular promise for addressing research questions that murine models imperfectly capture, such as liver-stage development, the biology of *P. vivax* hypnozoites, endothelial activation under flow conditions, sequestration dynamics, blood–brain barrier dysfunction, placental interactions, and tissue-specific drug responses. Beyond their translational potential, these innovative systems align with the 3Rs principles of Replacement, Reduction, and Refinement of animal experimentation, thereby reflecting both scientific rigor and ethical responsibility.

Despite these advancements, challenges persist in fully replicating the intricate biological of human malaria. For instance, the complete *Plasmodium* life cycle in vitro remains hindered by limitations in hepatocyte models. While immortalized hepatocyte lines offer advantages in continuous growth and genetic stability, they often exhibit reduced expression of drug-metabolizing enzymes and altered cellular architecture, leading to lower infection rates compared to primary hepatocytes. The HC-04 cell line represents a notable exception, demonstrating some capacity to support the hepatic stage, though it remains an imperfect surrogate. Moreover, the faithful recapitulation of the complex immune response in malaria infection within MPS, particularly regarding both cellular and humoral immunity, is still under development. While some progress has been made in generating rudimentary immune responses, the specificity and intricate mechanisms involving cell migration and cytokine storms observed in human pathology are not yet fully captured in current organoids or lymphoid node-on-chip systems. The integration of mechanical phenomena, such as clamping and patching, with MPS could further enhance the physiological relevance of these models in studying metabolic disruption and organ failure.

Therefore, the future of malaria research unequivocally lies in a multiscale, integrated framework (see Fig. 3). This approach will synergistically combine experimental power, genetic tractability, and practical accessibility of murine models with the physiological relevance and human-specific attributes of advanced human-based platforms. Such an integrative strategy demands better standardization of experimental readouts, stronger alignment between animal and human endpoints, broader utilization of genetically engineered parasites and hosts, and a closer integration of laboratory models with real-world clinical and epidemiological data from endemic regions. No single model, whether animal or *in-vitro* human-based, can fully replicate the immense complexity of human malaria. However, by strategically combining these complementary platforms rather than pursuing a singular, universal model, the field can achieve a more accurate, comprehensive, and translationally meaningful understanding of malaria biology. In this evolving scientific landscape, murine systems will continue to be indispensable, not as complete replicas of human disease, but as foundational components within a broader, dynamic experimental ecosystem, driving innovation toward improved prevention, diagnosis, and ultimately, the global eradication of malaria.

## Data Availability

All data produced in the present study are available upon reasonable request to the authors.

## Abbreviations

LT: T lymphocyte
LB: B lymphocyte
MΦ: Macrophage
ALT: Alanine transaminase
AST: Aspartate transaminase
BBB: Blood-Brain Barrier
BEI: Biodefense and Emerging Infections
BLTI: Block Lipid Transport inhibitor
CD34: Cluster of differentiation 34
CD36: Cluster of differentiation 36
CD8+: Cluster of differentiation 8+
CM: Cerebral malaria
ECM: Experimental cerebral malaria
EEC: Enteroendocrine
ENaC: Epithelial sodium channel
EPCR: Endothelial protein C receptor
FAH: Fumarylacetoacetate hydrolase
FRG: Immunocompromised and fumarylacetoacetate hydrolase-deficient
GPI: Glycosylphosphatidylinositol
HA: Primary human astrocyte
HBMEC: Human brain microvascular endothelial cells
HBVP: Primary human brain vascular pericytes
HC-04: Hepatoma cells
HCM: Human cerebral malaria
HIS: Human immune system
HO-1: Haem oxygenase
HSCs: Hematopoietic stem cells
huHep: Human Hepatocyte
HUVECs: Human umbilical vein endothelial cell
IB: Inbred
ICAM-1: Intercellular adhesion molecule
IL10: Interleukin 10
IL2rγ: Interleukin 2 receptor subunit gamma
iPSC: Induced pluripotent stem cell
iPSC-EC: Endothelial cells differentiated from human induced pluripotent stem cell line
IRB: Institutional review board
iRBC: Infected red blood cells
JAK/STAT: Janus kinase/signal transducer and activator of transcription
KO: Knock-out
LLINs: Long-lasting insecticidal nets
MA-AKI: Malaria-associated acute kidney injury
MA-ALI: Malaria-associated acute lung injury
MA-ARDS: Malaria-associated acute respiratory distress syndrome
MALP: Malaria-associated liver pathology
MHC: Major histocompatibility complex
MPS: Microphysiological system
MTT assay: (3-(4,5-Dimethylthiazol-2-yl)-2,5-diphenyl-2H-tetrazolium bromide) assay
NAFLD: Non-alcoholic fatty liver disease
ND: No data
NGAL: Neutrophil gelatinase-associated lipocalin
NHP: Non-human primate
NOD: Non-obese diabetic
OB: Outbred
OOC: Organ-on-a-chip
*Pb*: *Plasmodium berghei*
*Pb*A: *Plasmodium berghei* ANKA
*Pb*K173: *Plasmodium berghei* Keyberg 173
*Pb*NK65: *Plasmodium berghei* New York Katanga 65
*Pc*: *Plasmodium chabaudi*
*Pcc*: *Plasmodium chabaudi chabaudi*
*Pcc*AS: *Plasmodium chabaudi chabaudi* Avril-Scotland
*Pf*EMP1: *Plasmodium falciparum *Erythrocyte membrane protein 1
*Pg*: *Plasmodium gallinaceum*
*Pvk*: *Plasmodium vinckei*
*Pvp*: *Plasmodium vinckei petteri*
*Pvv*: *Plasmodium vinckei vinckei*
*Py*: *Plasmodium yoelii*
*Py*17XNL: *Plasmodium yoelii* 17XNL
*Py*YM: *Plasmodium yoelii* YM
Rag2: Recombination activating gene 2 protein
SCID: Severe combined immunodeficiency
SHIRPA: SmithKline Beecham, Harwell, Imperial College, Royal London Hospital, Phenotype Assessment
SMAC: Schizont membrane-associated cytoadherence
SNAP: Simple neuroassessment of asymmetric impairment
Th1: T helper cell type 1
TLR4: Toll-like receptor 4 (CD284)
UK: United Kingdom
USA: United States of America
VCAM-1: Vascular cell adhesion protein 1
WHO: World Health Organization
